# From Classical Genetics to Quantitative Genetics to Systems Biology: Modeling Epistasis

**DOI:** 10.1371/journal.pgen.1000029

**Published:** 2008-03-14

**Authors:** David L. Aylor, Zhao-Bang Zeng

**Affiliations:** 1 Bioinformatics Research Center and Program in Bioinformatics, North Carolina State University, Raleigh, North Carolina, United States of America; 2 Department of Genetics, North Carolina State University, Raleigh, North Carolina, United States of America; 3 Department of Statistics, North Carolina State University, Raleigh, North Carolina, United States of America; University of Alabama at Birmingham, United States of America

## Abstract

Gene expression data has been used in lieu of phenotype in both classical and quantitative genetic settings. These two disciplines have separate approaches to measuring and interpreting epistasis, which is the interaction between alleles at different loci. We propose a framework for estimating and interpreting epistasis from a classical experiment that combines the strengths of each approach. A regression analysis step accommodates the quantitative nature of expression measurements by estimating the effect of gene deletions plus any interaction. Effects are selected by significance such that a reduced model describes each expression trait. We show how the resulting models correspond to specific hierarchical relationships between two regulator genes and a target gene. These relationships are the basic units of genetic pathways and genomic system diagrams. Our approach can be extended to analyze data from a variety of experiments, multiple loci, and multiple environments.

## Author Summary

Epistasis has long had two slightly different meanings depending on the context in which it is discussed. The classical definition describes an allele at one locus completely masking the effect of an allele at a second locus. Such relationships can be interpreted as hierarchical, and they can be combined to infer genetic pathways. In quantitative genetics, epistasis encompasses a wide range of interactions and can be extended to more than two loci. These two definitions coexist because they are typically applied to different types of study populations and different types of traits. The current trend is to treat gene expression as a trait in a variety of genetic backgrounds. This provides reason to revisit epistasis in this new context. We accommodate the continuous nature of gene expression using ideas from quantitative genetics, but retain the hierarchical interpretation of the classical experiment. These hierarchical relationships are the building blocks of systems diagrams and genetic pathways. This framework can serve as a foundation for future epistasis analyses based on genomic data.

## Introduction

Epistasis has traditionally been discussed in two distinct contexts, corresponding to the disciplines of classical molecular genetics and quantitative genetics. In each case, the term describes an interaction between alleles at two or more loci. However, the methods for detecting epistasis and interpretations of the underlying biology have kept historical divisions in place despite calls for synthesis [1]. This is largely because the two fields traditionally study different types of traits in different experimental populations.

The classical epistasis experiment compares a double-mutant with two associated single-mutants. Epistasis is present if the observed double-mutant phenotype is categorized as being the same as a single-mutant phenotype. This implies a specific type of interaction in which an allele at one locus masks the effect of variation at the second locus. This relationship is described as the first locus being epistatic to the second, and can be interpreted as one gene acting upstream of the other. This hierarchical interpretation has been used to construct biological pathways via a series of epistatic gene pairs. However, this approach is limited by the necessity of easily observed and categorized phenotypes [2].

In contrast, quantitative genetics examines traits that vary continuously and cannot easily be categorized. Such trait distributions result from the cumulative effects of many genes. Each additional gene increases the possible combination of alleles, and the number of possible phenotypes grows exponentially. An individual's phenotype is the sum of the allelic effects at each gene and the effect of the environment. Epistasis is defined as a deviation from these additive gene effects [3]. A quantitative genetic model can include multiple loci and multiple interactions. Epistasis in this sense describes a functional relationship between genes in the context of a trait, but it includes both hierarchical relationships and nonhierarchical relationships and there is no way to distinguish between these.

Any genetic effect is only relevant to the population being studied due to the presence of genetic background. Background is genetic variation that is unobserved in the population and cannot be modeled. The classical experiment is performed using genetically homogenous laboratory strains so there is no background. Quantitative genetics studies diverse populations and background variation is almost always present. The implication of this is that epistasis may be detected in one experiment but not in another. This has led to criticisms that epistasis in the quantitative genetic sense is a statistical construct rather than a true representation of biology.

In fact, both approaches seek to illustrate underlying molecular architecture and each has its strengths. A hierarchical interpretation of epistasis is attractive as increased focus is placed on genetic pathways and systems diagrams. However, quantitative approaches are necessary to accommodate continuous data types such as gene expression, metabolite concentrations, and fitness. Recent literature suggests that such approaches are being adopted. For example, while early large-scale fitness profiles in yeast deletion mutants [4],[5] were scored categorically, St Onge et al [6] measured fitness in 650 double-deletion yeast strains and employed a novel quantitative analysis.

The rise in genomic techniques has broken down one of the traditional barriers discussed above: the same traits are now being used in both classical and quantitative settings [7]. Gene expression is perhaps the most prevalent example. Instead of a single phenotypic trait value, a vector of expression measurements describes each individual. Expression profiling in single-deletion yeast strains found that 34% of mutants showed twenty or more differentially expressed genes [2]. Expression quantitative trait locus (eQTL) mapping uses a linear modeling approach to associate genetic variation with gene expression traits [8]–[12]; Storey et al. [13] found over thirty percent of traits were jointly linked to two loci in yeast. When gene expression correlates with a complex phenotype, the corresponding traits may reflect the molecular basis of that trait at a level intermediate between genotype and phenotype. Some studies suggest that epistasis is pervasive among expression traits [14]–[16] and such traits may have more QTLs than classical traits [13],[17]. Since gene expression is being used in both classical and quantitative contexts, it is a valuable framework in which to compare the ability to detect epistasis and interpret the nature of relationships between genes.

We propose a framework for estimating and interpreting epistasis using expression traits. Our goal is to accommodate the continuous nature of the data, yet still preserve a hierarchical interpretation of epistasis. Such interpretations are well established for classical epistasis experiments [18], but have only recently been studied for complex data [19]. We refine the classical interpretations by explicitly modeling gene expression. Gene effects and interactions are estimated using a linear model, in a manner comparable to eQTL mapping. Our method selects the best-fit regression model for each trait, which describe the order and the nature of gene function. Such relationships are the basic units of genetic pathways and systems biology. We specifically address how to use a continuous phenotype in a manner that is both statistically sound and consistent with the classical approach.

We illustrate our method with publicly available expression measurements from *Dictyostellium discoideum* wild type [20] and deletion mutant strains [21]. This experiment is a classical epistasis analysis that targets the genes of the protein kinase (PKA) pathway and measures the gene expression profile of each strain.

## Results

### Modeling Epistasis for Continuously Variable Traits

In the classical epistasis analysis, triplets of deletion mutants combine with a wild type to form a contrast. Each contrast includes two single mutants and a double mutant. Each is described relative to the known wild type phenotype. A hypothetical example of a trait affected by two genes, *A* and *B*, can be described as follows, where *y* is the trait value, μ is the expected value of the wild type, β*_A_* and β*_B_* are the effects of deleting each gene, and ε is an error term.
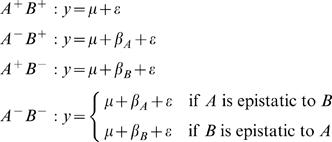



This adheres strictly to the classical definition, but there is a clear problem; there is no provision if the double mutant does not fall neatly into the same category as one of the single mutants. Gene expression traits fit poorly into the classical framework for this reason. Expression is continuous and intermediate levels are expected. Furthermore, even normalized trait values will inevitably include some measurement error. For these reasons, the double mutant observation is rarely the same as either of the single mutant observations or the wild type. Previous studies have attempted to circumvent this problem by relying on differences between the mutants to determine the most similar mutant pair. However, the assumption that expression is completely masked is poor. To address these issues, we move away from comparing trait values directly. Instead, we evaluate each deletion according to whether it significantly affects the expression of the target and associate unique patterns of significance with models of gene action.

We use a linear model to estimate the effect of each deletion. This is a general way to relate all mutants and the wild type without making any assumptions about the nature of the double mutant. We regress the trait value (e.g. expression) on indicator variables representing the presence or absence of each wild type allele and an interaction term. The interaction describes effects that are unique to the double mutant. The same example discussed above can be described as follows.

Trait value = Wild Type+Effect of deleting *A*+Effect of deleting *B*+Interaction+error
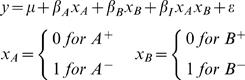



Various techniques can be used to fit such a linear model. We first fit a full model and then use stepwise backwards selection to drop model terms with coefficients that are not significant at a set level. The resulting reduced model is termed the best-fit model. For any trait, there are eight possible best-fit models. For clarity, we number the reduced models as follows:
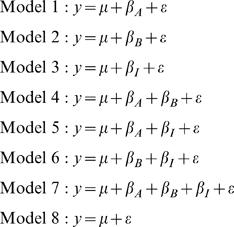



When the best-fit model has been determined, we estimate parameter values using that model for each trait. Thus, we have a best-fit model and coefficient estimates for each trait. The terms in each best-fit model represent the significant gene and interaction effects acting on that trait. Individual coefficients represent the estimated effect of deleting each gene. Model 7 corresponds to the classical model above when the interaction between the two deletions offsets the effect of one of them, either *β_I_* = −*β_A_* or *β_I_* = −*β_B_*. Model 8 describes the case in which the deleted loci have no effect on the trait.

A best-fit model describes each gene expression trait. As such, we have dealt with the continuous variable problem. However, by embracing a quantitative genetic model we have lost the appealing feature of the classical experiment: the ability to interpret hierarchical relationships. In the following section we identify sixteen hierarchical relationships and propose that a specific best-fit model supports each.

### Interpreting Hierarchical Epistasis

In quantitative genetics, the interaction term in the above model is considered epistasis. However, epistasis in this sense includes both hierarchical and nonhierarchical relationships. Conversely, while Model 7 can clearly be interpreted as hierarchical epistasis with the conditions described above, it does not apply to all possible hierarchies.

**Figure 1:**
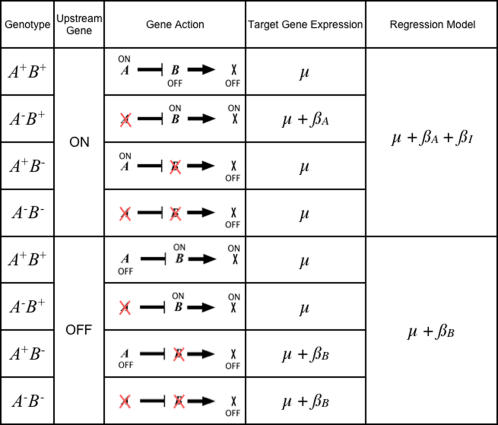
Modeling the Relationship *A* Is an Upstream Repressor of *B*. *B* in Turn Enhances a Target Gene *X*. In this example, deleting *A* will change the state of the target gene from off to on. Therefore, we include *A*'s effect in the corresponding regression model. Deleting *B* leaves the target gene in the same state as the wild type and its effect is not included. The *AB* double mutant is also not expected to deviate from the wild type despite the significance of the *A* deletion. Since *A*'s effect is already included in the model for this contrast, it must be offset by the interaction term. We conclude that if *A* is enhanced by the signal, *A* represses *B*, and *B* enhances *X*, the corresponding best-fit regression model will include coefficients for *A* and an interaction term. Similar logic applies to the case in which the signal represses *A*. The signal represses *A*, thus deleting *A* has no downstream effects. We expect only the coefficient corresponding to the downstream gene in the best-fit model.

We considered all combinations of gene order and action within simple ON/OFF models and then predicted the hypothetical effect of deleting genes on each of them (Figure 1, Figures S1, S2, and S3). There are four points of variation to model for each gene pair relationship. The first is the identity of the upstream gene, i.e. the gene order. Secondly, the upstream gene will turn the downstream gene either on (enhance) or off (repress). Thirdly, the downstream gene can enhance or repress the expression of a target gene for which expression is observed. Lastly, we consider that the upstream gene itself will be enhanced or repressed by some initiating factor such as a developmental cue or environmental perturbation. Avery and Wasserman [18] provide a general framework that has been widely used for interpreting epistasis in response to such signals, and note that the effect of a mutation is only observable for a specific signal state. However, knowing the signal state does not give any information about whether the upstream gene is enhanced or repressed in that state. In our models, we focus on the effect on the upstream gene. This model has sixteen possible variants describing hierarchical relationships between two genes and the target gene.

The key to our approach is connecting each of the sixteen hierarchical models to one of the eight possible best-fit regression models. If the deletion changes the state of a target gene relative to the wild type in a mutant, then that deletion is predicted to have a significant effect and it will be included in the regression model corresponding to that hierarchical model. Figure 1 gives an example of one possible model, in which *A* is enhanced by a signal; *A* is an upstream repressor to *B*; and *B* enhances a target gene *X*. We conclude that the corresponding best-fit regression model will include coefficients for *A* and an interaction term. Note that if the signal instead represses *A*, a different best-fit model represents the same relationship between *A* and *B*.

We applied the same approach to each of the sixteen cases and note several trends. First, the downstream gene's effect upon the target gene *X* does not influence the corresponding best-fit model. This allows us to reduce the model space to eight hierarchical relationships (Table 1). This observation is convenient, because expression traits represent all the genes downstream of the deletions. Regardless of the downstream gene's direct effect, some traits will be enhanced while others are repressed. When the upstream gene is a repressor, four distinct regression models represent four unique hierarchical relationships. We can uniquely identify both the gene order and signal effect on the upstream gene. We cannot discern gene order if the upstream gene is an enhancer because the same best-fit model describes both hierarchies. If the upstream gene is merely enhancing the effect of the downstream gene, deleting either gene will affect the trait gene similarly. Six of the eight possible best-fit regression models correspond to the eight hierarchical relationships. It is notable that hierarchies can be indicated even without an interaction effect in the model.

**Table 1:** Correspondence Between Regression Models and Biological Models.

a. Hierarchical Relationships				
	A upstream of B		B upstream of A	
Upstream Gene	ON	OFF	ON	OFF
Repressor	μ*+*β*_A_+*β*_I_* [5]	μ*+*β*_B_* [2]	μ*+*β*_B_+*β*_I_* [6]	μ*+*β*_A_* [1]
Enhancer	μ*+*β*_A_+*β*_B_+*β*_I_* [7]	μ [8]	μ*+*β*_A_+*β*_B_+*β*_I_* [7]	μ [8]

b. Non-hierarchical Relationships				
State of A/B	ON/ON	ON/OFF	OFF/ON	OFF/OFF
Enhancer/Enhancer	μ*+*β*_I_* [3]	μ*+*β*_A_* [1]	μ*+*β*_B_* [2]	μ [8]
Enhancer/Repressor	μ*+*β*_A_+*β*_B_* [4]			
Or				
Repressor/Enhancer				
Repressor/Repressor	μ*+*β*_I_* [3]			

**a.** Six of the eight possible regression models represent hierarchical relationships between genes. If the upstream gene is a repressor we can identify gene order and the signal effect. If the upstream gene is an enhancer, we can identify only the signal effect. If the signal turns off an upstream enhancer, deleting either gene will have no effect. **b.** Non-hierarchical relationships can be distinguished if both genes are activated by the signal. Model 3 suggests buffering, while Model 4 suggests independent effects, i.e. no epistasis. If a potential regulator is turned off by the signal it has no effect on the target gene.

We must also consider that there is no hierarchical relationship between *A* and *B*, or that they do not affect the target gene (Table 1). We can distinguish between two types of parallelism. Model 4, the two-gene additive model with no interaction, represents no epistasis. Model 3 represents buffering epistasis, in which both genes act on the target in the same direction, and the effect of deleting either is not apparent unless both genes are deleted. We refer to this as nonhierarchical epistasis since neither gene is upstream of the other. Deleting a deactivated regulator gene has no effect on the target gene, making it impossible to identify a biological relationship when regulators are deactivated.

The remainder of Table 1 represents cases in which one or both genes do not affect the target gene. Expression traits supporting Model 8 (no significant terms) may represent target genes that do not lie downstream of *A* or *B*, and are uninformative. The result is one-to-many relationships between best-fit regression Models 1, 2, and 8 and their corresponding gene expression models. If the upstream gene of a hierarchical pair is turned off, we cannot know whether it is upstream or uninvolved.

Typically, expression is measured from thousands of genes simultaneously and we do not expect them all to be informative. Even with clear interpretations for each trait individually, there is a challenge interpreting all traits together. We examine the distribution of all traits. Among informative traits associated with a best-fit model, the majority may represent the underlying biological relationship between the deleted genes.

### Validating the Two-Step Modeling Framework

Van Driessche et al. used *Dictyostellium discoideum* wild type [20] and deletion mutant strains [21] to infer hierarchical epistasis among genes of the protein kinase (PKA) pathway. Each strain's gene expression profile was measured using cDNA microarrays with a common reference over 24 hours. These data are well suited for testing our methods for two reasons. First, the epistatic relationships between the deleted genes already have been characterized experimentally. Secondly, the mutant strains are genetically identical at all loci except the few being studied, i.e. there is no variation in their genetic background.

The PKA pathway is associated with the developmental aggregation response to nutrient deprivation, which initiated midway through the time course. Data before and after aggregation were considered separately so we can clearly interpret the deletion effects in each signal state. The data represented fold-change on a logarithmic scale, which made the distribution of expression measurements approximately normal; we consider the implications of this in the discussion. We studied 1553 expression traits. The genes we used were measured in both experiments and differentially expressed in the wild type during aggregation [20]. Five deletion strains target genes of the protein kinase A (PKA) pathway that is involved in the response to starvation and activates aggregation. This provided three contrasts: *pufA*/*pkaC*, *pufA*/*yakA*, and *regA*/*pkaR*. Although there are ten possible contrasts for these five genes, only these three double mutants were generated, presumably because these are known direct relationships.

**Figure 2:**
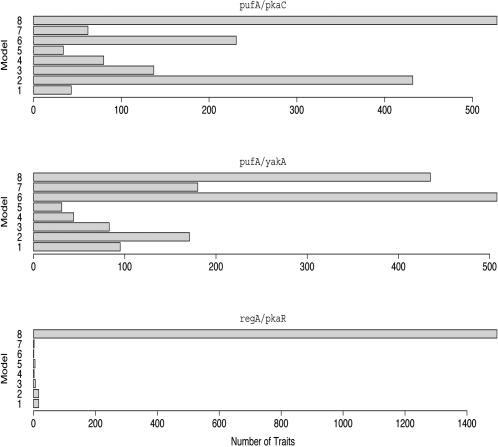
Post-Aggregation Distribution of Best-Fit Models at p<0.01 Significance Threshold. The frequency distribution of best-fit regression models can be interpreted as hierarchical relationships between genes. Model 8 corresponds to no deletion effects and is supported by a large number of traits in each contrast; these genes are likely not downstream of the deletions. The model supported by the majority of remaining traits is assumed to represent the true relationship.

For each contrast, some traits supported each model (Figure 2). Additionally, large number of traits showed no deletion effects (i.e. support Model 8). At a significance threshold of p<0.01, a majority of traits supported Model 8 for every contrast pre-aggregation (Figure S4) and for the *regA*/*pkaR* contrast post-aggregation. According to our interpretive models, Model 8 can indicate three possibilities. The first two are hierarchical relationships in which an upstream enhancing gene is turned off during aggregation. The last possibility is that the genes are uninvolved in the expression of the target and the deletions have no effect.

Since not all target genes are downstream of the PKA pathway, it is logical that the deletions have no effect on these genes. Similarly, the PKA pathway is invoked during aggregation and it follows that the deletions may affect expression only after aggregation has begun. We assume that the target genes supporting Model 8 are not downstream of the pathway, and that the majority of the remaining target genes reflect the relationship within the pathway. To test this assumption, we looked at the overlap between the expression traits supporting Model 8 for each contrast. We found that all of the expression traits supporting Model 8 for the *pufA*/*yakA* contrast also supported Model 8 for the other two contrasts. These traits strongly support the assumption that they are not downstream of the PKA pathway.

When we looked at these genes for both the *pufA*/*pkaC* and *pufA*/*yakA* contrasts, there was strong support for one model over all others post-aggregation. Not only did these models explain more traits post-aggregation, but the models also fit better. On average, the best-fit model explained over half of the expression variation (R^2^≥0.5, adjusted for degrees of freedom in the model) for traits in the *pufA*/*pkaC* and *pufA*/*yakA* contrasts, and for both contrasts the R^2^ increased post-aggregation (t-test with p<0.0001).

For the *pufA*/*pkaC* contrast, Model 2 had the most support of the seven non-null models. Model 2 corresponds to two possible interpretations. The first is that *pkaC* is the downstream gene, that *pufA* is a repressor, and that the *pufA* is turned off in the presence of the aggregation signal. Alternately, we could interpret it to mean that only *pkaC* has an effect on the downstream targets and that *pufA* is unrelated. For the *pufA/yakA* contrast, Model 6 had the most support among non-null models. This model has a one-to-one correspondence to our interpretive models. It asserts that *yakA* is an upstream repressor of *pufA*, and that *yakA* is turned on at aggregation. These conclusions both agree with what has been determined previously about the roles these three genes play during development [22]. *YakA* represses *pufA*, which then ceases to repress *pkaC*.

The *regA/pkaR* was problematic because almost all traits supported Model 8, the model with no effect terms. For the previous two cases, we assumed that these traits were not downstream of the pathway. Given this assumption, we could have concluded that *regA* and *pkaR* were not involved with aggregation. However, the other two contrasts had 435 and 528 traits supporting Model 8, while *regA/pkaR* has 1497. Because of this discrepancy, we suggest that some proportion of these genes support the hierarchical model corresponding to Model 8: that one gene is an enhancer of the other and is deactivated by aggregation. According to previously published results, *regA* and *pkaR* work together to repress *pkaC* pre-aggregation and are in fact deactivated post-aggregation [23]. This is consistent with the potential hierarchical relationship.

Because we are modeling nonadditive interactions, the logarithmic scale transformation on these data can potentially alter the results relative to untransformed data [3],[24]. To test this, we exponentiated the data and repeated our method. Despite dramatic changes to the shape of the data distribution, the resulting distribution of best-fit models agreed with the results presented above. Again, a majority of traits showed no deletion effects (i.e. support Model 8). Model 2 had the most support for the *pufA*/*pkaC* contrast, Model 6 had the most support for the *pufA*/*yakA* contrast, and Model 8 had near complete support for the *regA/pkaR* contrast using the post-aggregation data (Figure S5). Interestingly, this does not imply that each trait supports the same model regardless of the scale transformation. In fact, only 57% and 47% of traits support the same model with the untransformed data for the *pufA*/*pkaC* contrast and *pufA*/*yakA* contrast respectively. However, in both these cases the vast majority of changed traits support Model 8. This result amends our previous interpretation of the traits supporting Model 8; in addition to genes not downstream of the pathway, there may be some proportion of genes for which expression changes due to deletion is not detectable due to issues of scale. Fewer traits supported Model 8 using transformed data, suggesting that these data may be more informative using the logarithmic transformation.

Thus, in all three cases our best-fit regression models correspond to a set of interpretative models that includes the true relationship between the genes. Certain regression models have a one-to-many relationship with the interpretive models, but in these cases the number of candidate interpretive models is reduced to a few. Only one interpretation corresponds to Model 6, which makes the *pufA*/*yakA* contrast straightforward to describe. In evaluating *pufA*/*pkaC*, Model 2 corresponds to one hierarchical model and one single-gene model. Since the *pufA*/*yakA* contrast provides evidence that deleting *pufA* has an effect, the hierarchical model is a preferable interpretation to the *pkaC* only model. As we vary the significance threshold for model selection, our results are robust. The best-fit model among models 1–7 was the same for p-value thresholds from 0.05 to 0.001 (Figure S6). As the selection criterion becomes stricter we reject more effects as not significant, and more traits support Model 8.

## Discussion

Measuring transcript abundance within a cell will remain a fundamental interest to biologists. Gene expression technologies have become popular over the past decade because of their ability to capture many genes simultaneously. Analyses that traditionally focused on a few genes now must be expanded to consider entire genomes. At this scale, the relationships between genes are of as much interest as the genes' individual effects. Many methods exist to infer gene networks or pathways from expression profiles [25]. Most of these require large datasets and result in large network diagrams that are difficult to interpret. These approaches are useful because they provide a genome scale view of transcription, and they are convenient because they can be applied to data from a variety of easily accessible sources.

However, there is a continuing need for experiments that allow us to infer pathways directly. The classical epistasis experiment we recount in our results [21] is one such approach. Because it targets gene pairs directly, we can build pathways a relationship at a time. This local approach results in pathway diagrams that are easily comprehended and biologically relevant. Additionally, it associates genetic variation with expression variation. For these reasons, these types of experiments will be increasingly useful in constructing biological systems diagrams. While there are currently few experiments that measure expression in a genetically variable population, their number is increasing rapidly. Our motivation is to provide a conceptual framework in which these and related experiments can be interpreted. We have addressed the simplest genetically variable data structure for identifying epistasis, in which individuals vary at only two loci, but our ideas can be applied to a range of similar data.

Because expression data are continuous by nature, we must address them with quantitative methods. Regression analysis is a standard technique to relate continuous variables. Using a multiple regression model to estimate gene effects and interactions has several advantages. First, it allows us to consider information from all the deletion mutants and the wild type simultaneously. Additionally, it estimates an effect for each allele, allows for variance in allelic effects, and separates these effects from error variance. In a traditional epistasis analysis the double mutant is compared to each single mutant in a rule-based manner, and the two nearest trait values determine epistasis. In contrast to our method, this method does not take advantage of all the information from a given contrast, and it is difficult to distinguish signal from noise. Myriad sophisticated techniques exist for fitting multiple regression models, and these should be employed based on the distributional properties of particular data.

We consider individual expression traits rather than an expression profile. A gene expression model represents each trait, but we must infer the correct biological model through the results from the regression step. A corresponding regression model represents each possible gene expression model, but these relationships are not always one-to-one. Hierarchies in which an upstream gene is turned off by a signal are confounded with cases in which the gene has no effect. It makes sense that we cannot observe the effect of a deletion if the gene is already turned off in the wild type. Nonetheless, our framework was consistent with previous characterizations of the pathway in every case.

Scale transformations are common in genetics and genomics so that data meet statistical testing assumptions such as normality and homoscadasity [3]. Logarithmic transformations are ubiquitous in the literature for gene expression data such as that presented in our results. However, models with nonadditive interactions are subject to the scale of the data, and transformations can result in support for alternative models. This is a long-standing problem with describing epistasis for complex traits [24]. Often it is difficult to know the most biologically appropriate scale, and the scale is instead often chosen arbitrarily based on the available measurement or statistical convenience. For gene expression traits the scale issue is even more complex. Since there are wide differences in the range of expression variation between genes, it is likely that no one scale will allow detection of the underlying biological interactions for all expression traits. The relationship between scale and epistasis is an area that demands further study, particularly in this era of genetics on biomolecular traits such as gene expression that have not been well studied in this context.

When we performed the same analysis on log-transformed and untransformed post-aggregation data, about half the traits supported a different best-fit model, yet the distribution of results led to the same conclusions regarding the underlying relationship between the deleted genes. This suggests our conclusions may be robust to scale effects that would affect single traits because they are based on the distribution of all traits. Those traits that are affected by scale trend toward having no detectable deletion effects with untransformed data. This further confounds the roughly one-third of traits supporting Model 8, which may also suggest an upstream enhancer or a trait truly unaffected by the deletions. While we do not discount scale effects, we assume most of these traits fit the last category because of the high percentage of these traits, the consistency of traits supporting Model 8 between contrasts, and the logic that deletions should affect only downstream genes. Whichever the case, these concerns make a strong argument for interpreting the distribution of results across expression traits. This contrasts with methods that consider all traits as an expression profile. These assume the profile as a whole supports one underlying pathway [21].

Using our method, it is straightforward to interpret a range of experiments. The alleles being studied do not need to be null alleles, e.g. deletions. The same method could be applied to over-expressed genes, or any polymorphic locus. Additionally, the method can accommodate experiments investigating multiple loci and higher order interactions. Three-way and four-way epistasis models follow from the same principles as the two-way models we present. The regression model is very flexible and easy to extend by adding a parameter for each locus plus interaction terms. Connecting these statistical models to biological models follows the same process we have illustrated. The strengths of our approach are particularly apparent in multi-locus models because we provide a means for estimating effects using the entire population of mutants simultaneously. The number of genotypes increases by a power of two for each additional gene included in the experiment; with a three-locus experiment having eight genotypes. As the number of necessary pair-wise comparisons increases, they will contain more undetected error and become more difficult to interpret. Environmental effects can also be included in the model at the expense of increased complexity in interpretation. We considered observations before aggregation and after aggregation separately in our example for simplicity.

By proceeding to add genetic and environmental complexity, it is apparent how the classical epistasis framework connects to the quantitative genetic paradigm. An additional benefit of our method is that it enables comparisons between any population-based expression analyses. Whether study populations consist of deletion mutants, experimentally designed crosses, inbred lines, chromosome substitution strains, or natural populations, each expression trait is the same. For this reason, comparing these results is highly desirable. Estimating the allelic effects and interactions for each expression trait allows direct comparison across a variety of genetic backgrounds. By embracing a common interpretive framework to a range of experiments that use gene expression as a trait, we can integrate results and form clearer insights into the genetic control of systems.

## Materials and Methods

### Dictyostellium Gene Expression Data

We used data originally presented by Van Driessche et al. We use data from *Dictyostellium discoideum* wild type [20] and eight deletion mutant strains (*pufA^−^, pkaC^−^*, *pufA^−^pkaC^−^*, *yakA^−^*, *pufA^−^yakA^−^*, *regA^−^, pkaR^−^, regA^−^pkaR^−^*) [21]. They measured each strain's gene expression profile over a time course using cDNA microarrays and a common reference that was pooled from all time points. Expression was measured thirteen times over 24 hours and captured the developmental aggregation response to nutrient deprivation, which initiated midway through the time course. We grouped observations before (hours 0,2,4,6) and after (hours 14,16,18,20) aggregation. Expression at these time points is highly correlated ([Figure 2 in 20]) and consistent with the regulatory changes previously reported. This data pooling increased the sample size for our regression analysis. Observations during the transitional period (hours 8,10, and 12) were disregarded, as were observations in the late stages of development that were less correlated (hours 22 and 24). The data represented fold-change on a logarithmic scale. We studied 1553 genes that were measured in both experiments and differentially expressed in the wild type during aggregation [20].

### Regression Analysis

We fit models in the R statistical environment [26]. Stepwise backwards selection entails fitting a fully parameterized model, then eliminating model terms that do not meet a specified significance threshold. The model is refit with the remaining terms until no further terms can be dropped.

## Supporting Information

Figure S1 Modeling the Relationship A is an Upstream Repressor of B, which Represses a Target gene.
(0.07 MB TIF)

Figure S2 Modeling the Relationship A is an Upstream Enhancer of B, which Represses a Target Gene.
(0.07 MB TIF)

Figure S3 Modeling the Relationship A is an Upstream Enhancer of B, which Enhances a Target Gene.
(0.07 MB TIF)

Figure S4 Distribution of Best-Fit Models Pre-Aggregation.
(3.65 MB TIF)

Figure S5 Distribution of Best-Fit Models Post-Aggregation (Untransformed Data).
(3.61 MB TIF)

Figure S6 Distributions of Best-fit Models at Varying Significance Thresholds
(0.28 MB XLS)
