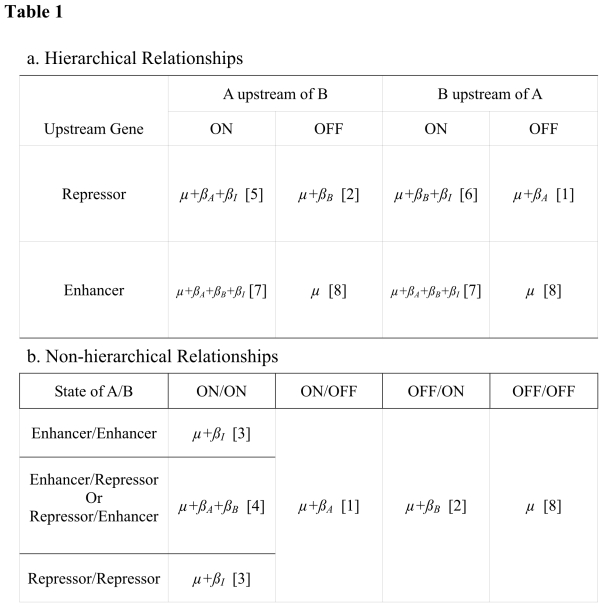# Correction: From Classical Genetics to Quantitative Genetics to Systems Biology: Modeling Epistasis

**DOI:** 10.1371/annotation/ff93eba8-9567-4f41-b90d-9cdfdf65f747

**Published:** 2008-05-19

**Authors:** David L. Aylor, Zhao-Bang Zeng

Table 1b is not displayed correctly in the published article. Please see the correct Table 1 here:

**Figure:**