# Supplementary figures and images for: From Classical Genetics to Quantitative Genetics to Systems Biology: Modeling Epistasis
Source: PLoS Genet. 2008 Mar 14;4(3):e1000029. doi: 10.1371/journal.pgen.1000029 (PMC2265472; doi:10.1371/journal.pgen.1000029)

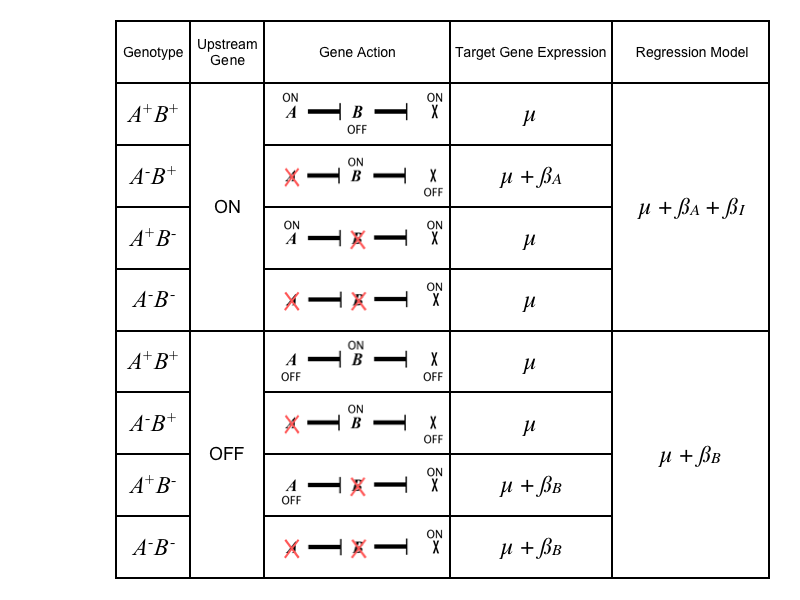

Supplement: Figure S1 — Modeling the Relationship A is an Upstream Repressor of B, which Represses a Target gene. (0.07 MB TIF) [file pgen.1000029.s001.tif]

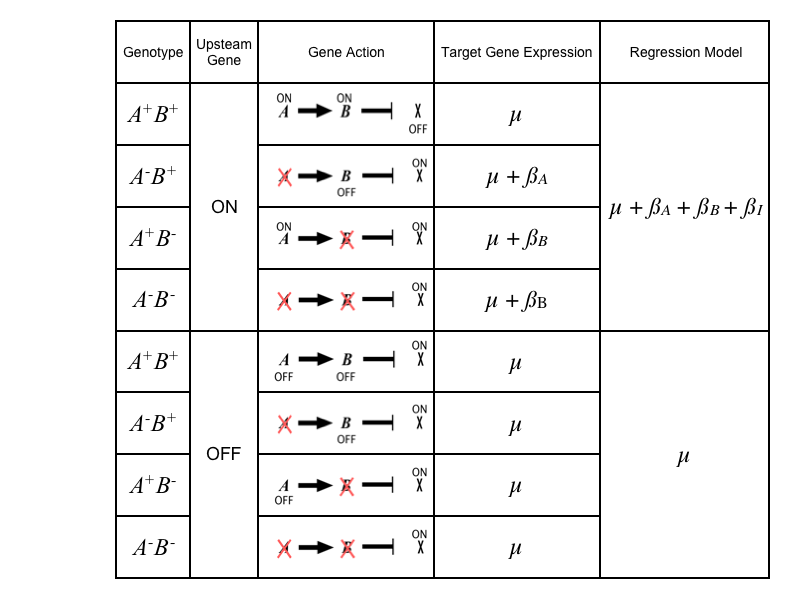

Supplement: Figure S2 — Modeling the Relationship A is an Upstream Enhancer of B, which Represses a Target Gene. (0.07 MB TIF) [file pgen.1000029.s002.tif]

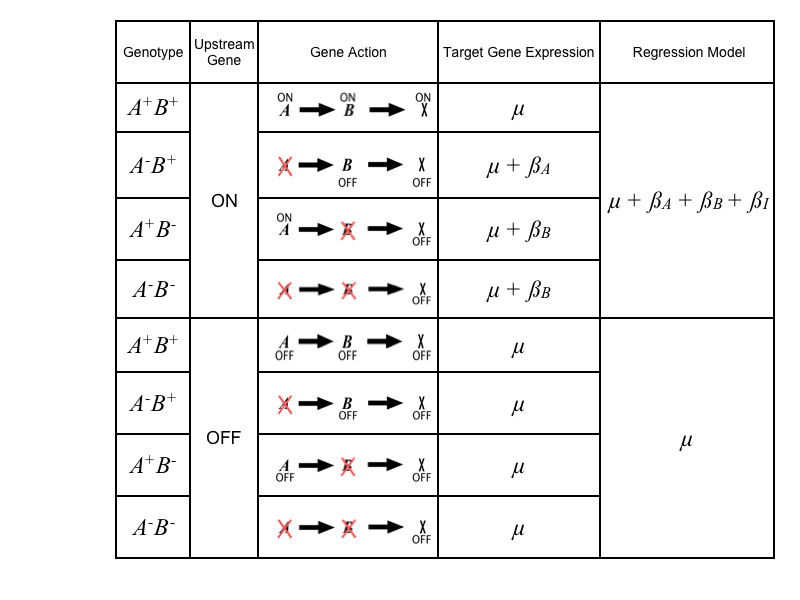

Supplement: Figure S3 — Modeling the Relationship A is an Upstream Enhancer of B, which Enhances a Target Gene. (0.07 MB TIF) [file pgen.1000029.s003.tif]

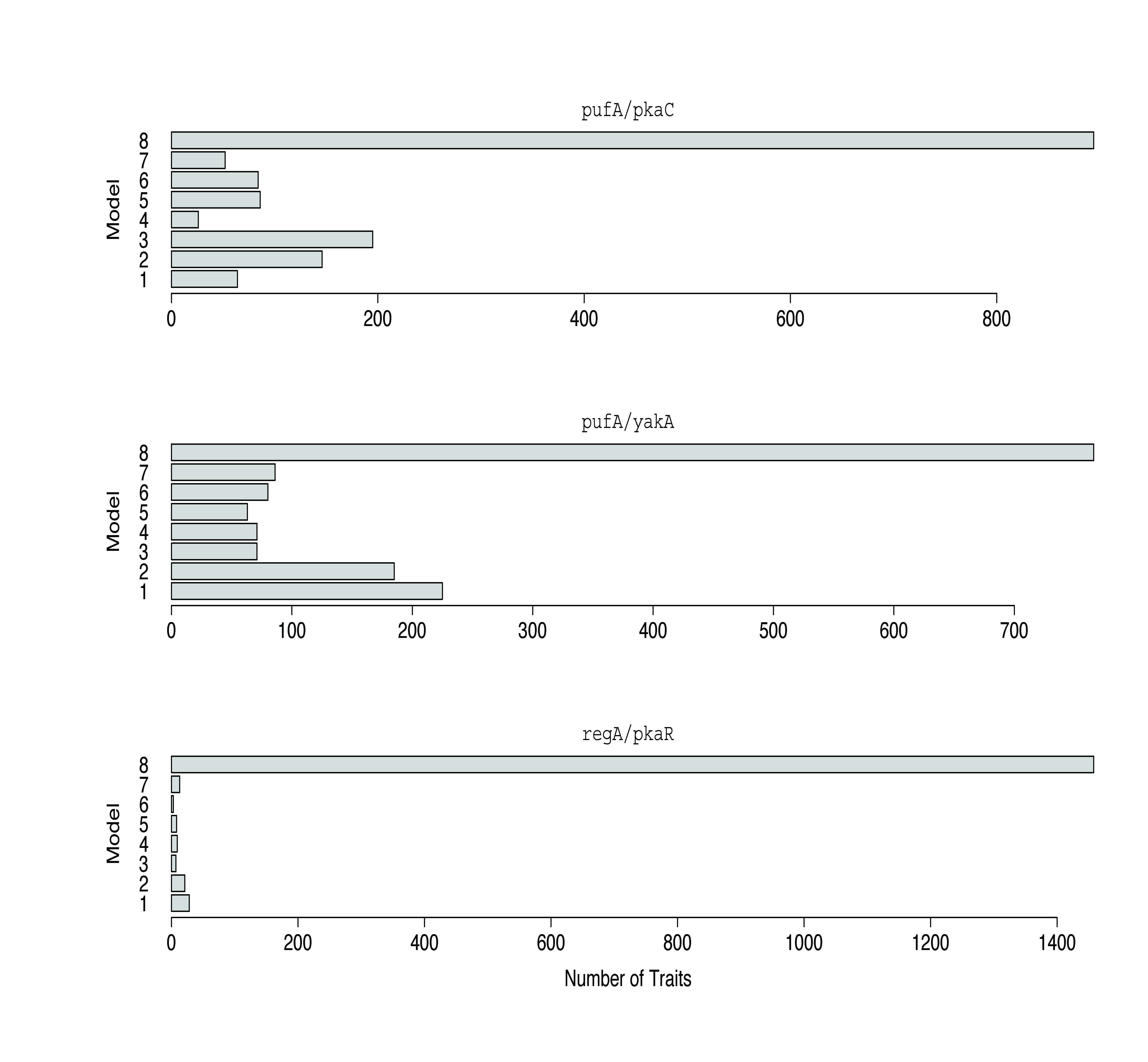

Supplement: Figure S4 — Distribution of Best-Fit Models Pre-Aggregation. (3.65 MB TIF) [file pgen.1000029.s004.tif]

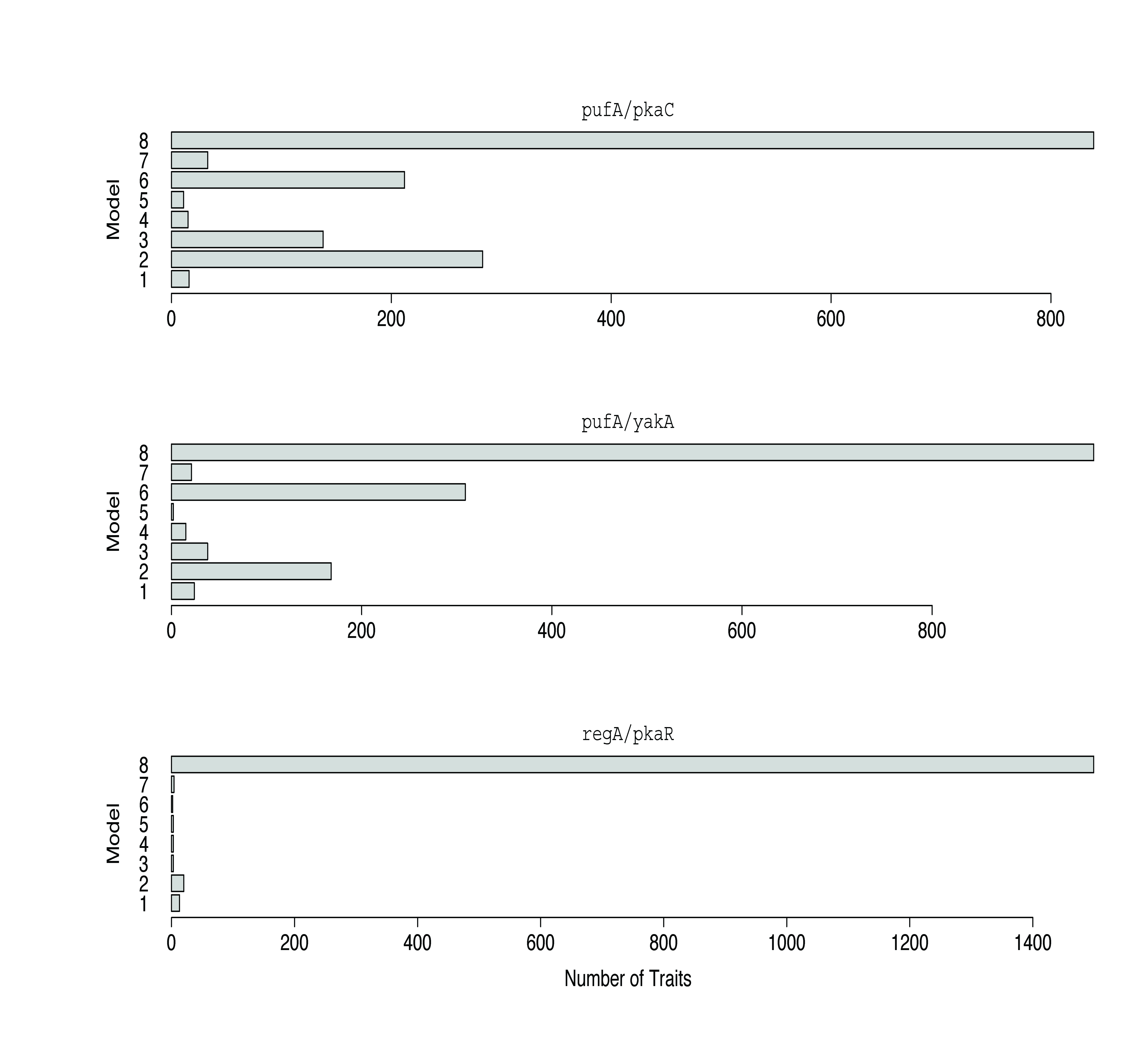

Supplement: Figure S5 — Distribution of Best-Fit Models Post-Aggregation (Untransformed Data). (3.61 MB TIF) [file pgen.1000029.s005.tif]
